# Case Report: Diffuse Cerebral Microbleeds in Cerebral Autosomal Recessive Arteriopathy With Subcortical Infarcts and Leukoencephalopathy

**DOI:** 10.3389/fneur.2022.818332

**Published:** 2022-02-09

**Authors:** Lan Wen, Jichao Yuan, Shuang Li, Jieyi Zhao, Congjun Li, Jiafei Li, Yuanyuan Han, Chaohua Wang, Guangjian Li

**Affiliations:** ^1^Department of Neurosurgery, West China Hospital, Sichuan University, Chengdu, China; ^2^Department of Neurology, Southwest Hospital, Third Military Medical University (Army Medical University), Chongqing, China; ^3^Department of Neurology, The Affiliated Hospital of Southwest Medical University, Luzhou, China

**Keywords:** CARASIL, cerebral microbleed, *HTRA1* mutation, leukoencephalopathy, cerebral small vessel disease

## Abstract

Cerebral autosomal recessive arteriopathy with subcortical infarcts and leukoencephalopathy (CARASIL) is a hereditary cerebral small vascular disease caused by a homozygous mutation in the high-temperature requirement A serine peptidase 1 (*HTRA1*) gene. Cerebral microbleeds (CMBs) are increasingly being recognized as neuroimaging findings occurring with cerebrovascular disease and have different etiologies. Mild to moderate CMBs are not unusual in CARASIL, and they are observed to affect cortical and subcortical structures; in contrast, diffuse CMBs, especially in the cerebellum, are rare. In this case, we report a novel mutation of *HTRA1* in a 43-year-old woman whose imaging indicated multiple CMBs in all lobes, brain stem, and cerebellum. The amount and location of CMBs vary in CARASIL cases, and the potential cause is not fully understood. This study revealed that specific imaging findings of this patient may be related to a new genetic mutation.

## Introduction

Cerebral autosomal recessive arteriopathy with subcortical infarcts and leukoencephalopathy (CARASIL) is a very rare autosomal recessive non-hypertensive cerebral small vessel arteriopathy caused by biallelic mutations of the high-temperature requirement protease A1 (*HTRA1*) gene. Additionally, it is characterized by subcortical infarcts, alopecia, and spondylosis (1). Furthermore, leukoencephalopathy with multiple lacunar infarctions, brain atrophy, and cerebral microbleeds (CMBs) have been observed on magnetic resonance imaging (MRI) in previous studies (2–4). Presently, there are very few studies on CMBs in CARASIL, most of which are reported to be located in the cortex and subcortex, mainly in the basal ganglia region (5–9). Herein, we report a 43-year-old woman with a novel *HTRA1* gene mutation who presented widely distributed CMBs in the brain lobes and deep regions, especially the cerebellum.

## Case Presentation

**Figure 1:**
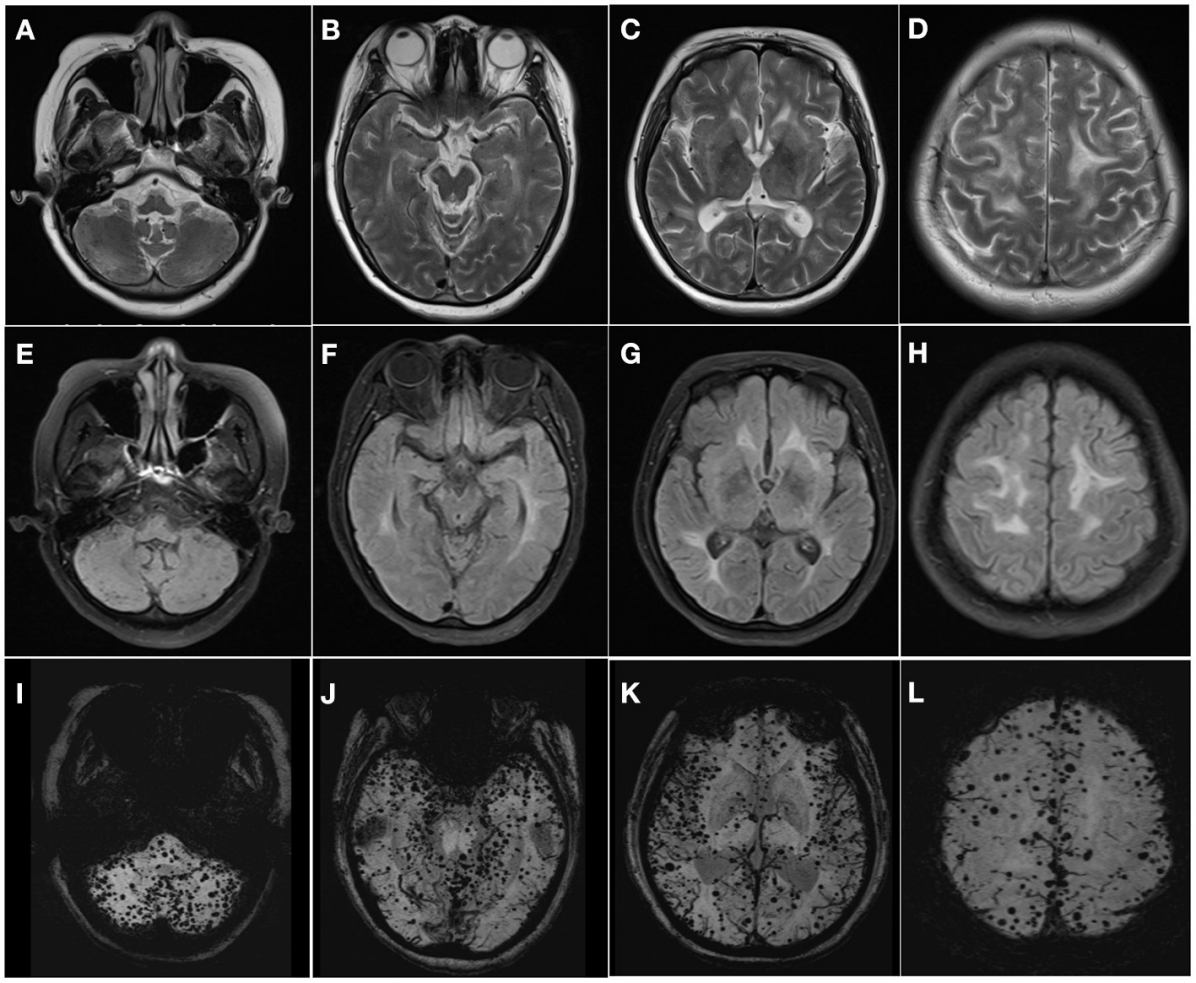
Multiple hyperintensities in the cerebral white matter extending from supra- to infratentorial regions on T2-weighted **(A–D)** and fluid-attenuation inversion recovery images **(E–H)**. Susceptibility-weighted imaging shows diffuse microbleeds in the cerebellum, brain stem, subcortex, and cortex **(I–L)**.

The proband was a 43-year-old woman with progressive gait disturbance and memory impairment for 1 year and inarticulate speech for 2 months. One year before admission, she developed walking instability and slight memory loss without any regular treatment. Since the last 2 months, slurred speech and comparatively obvious cognitive impairment have been observed. Additionally, the patient had no hypertension, diabetes, or other diseases except for mild hair loss at a young age. The positive sign of physical examination were mild alopecia, unsteady walking, ataxic gait, and inarticulate speech, accompany with moderate cognitive impairment and Mini-Mental State Exam score of 18 points (illiteracy), demonstrating majorly focused on orientation, memory, and computed function. Further neurological examination revealed normal muscle strength and tone, whereas exhibited positive bilateral Babinski signs. Moreover, she failed to complete the dysmetria and dysdiadochokinesia and underwent the Finger to-Nose test. MRI revealed white matter hyperintensities in the periventricular centrum semiovale and brain stem on T2-weighted and fluid-attenuation inversion recovery images; additionally, extensive microbleeds were observed in the frontal, temporal, and occipital lobes, brain stem, and cerebellum on magnetic susceptibility-weighted imaging (Figure 1). The imaging showed leukodystrophy and extensive CMBs tending toward cerebral small vascular disease (cSVD), which appeared similar to cerebral amyloid angiopathy (CAA) at first glance. However, deep CMBs in the cerebral and cerebellar regions were similarly observed; in contrast, other CAA-related features, such as convexity subarachnoid hemorrhage and cortical superficial siderosis, were absent. To further clarify the diagnosis, vasculitis markers were assessed, and angiography was performed, both of which showed negative findings. The blood pressure was noted an average at 136/80 mmHg. Considering the patient's hair loss, which is a symptom of CARASIL, we detected a genetic mutation linked to hereditary cSVD, and targeted panel-sequencing revealed a novel homozygous missense mutation (c.508 A>C, p.N170H, chr10:124248453) in the *HTRA1* gene, which exhibited heterozygous mutation in her father. Finally, the diagnosis was confirmed as CARASIL. Following the administration of donepezil and some neurotrophic treatments, the patient's symptoms did not improve but progressively worsened. At six months of follow-up after discharge, she could neither walk nor independently perform daily living activities.

## Discussion

CARASIL is an extremely rare cSVD, with most cases reported in Asia, mainly in Japan and China. A few reports on Caucasian patients have also been published in recent years (9–11). The clinical manifestations include early adult-onset subcortical infarcts, progressive motor and cognitive impairment, alopecia, and spondylosis; non-neurologic symptoms include special signs that distinguish CARASIL from other diseases (1, 3, 4). Due to the *HTRA1* mutation, dysregulation of the inhibition of signaling by members of the TGF-β family induces cerebral small vessel disease and has also been linked to alopecia and spondylosis. The central pathology involves the lack of vascular smooth muscle cells (VSMCs) in the media and adventitial fibrosis, which invades the small penetrating arteries and mainly affects the cerebral white matter and basal ganglia (12, 13). Loss of VSMCs is the primary feature of CARASIL, followed by deposition of granular materials in the media and fibrosis of the arterial wall, leading to ischemic and hemorrhagic consequences; consequently, diffuse white matter hyperintensity, lacunar infarctions, CMBs, and encephalatrophy are observed on MRI (14).

CMBs are increasingly being recognized as a characteristic marker of cSVD on neuroimaging and progressing in various locations for specific vascular pathologies among different etiology. For example, the lesions in leptomeningeal and cortical vessels contribute to strict lobar distribution, suggesting CAA, while the deep perforating arteries match with the hypertensive vasculopathy. Since the pial arteries, perforating arteries, and arterioles are affected in CARASIL, the lobes and deep brain regions are potentially involved in the pathologies simultaneously (4). Previously, mild to moderate CMBs were observed in patients with partial CARASIL, preferentially involving the juxtacortical hemispheric areas, thalamus, and brain stem (5–9). This patient showed multiple CMBs in all lobes, especially in the cerebellum, which is rarely reported. Since other vascular risk factors were absence, and the imaging findings, lacunar infarctions and atrophy, were less severe, such numerous CMBs and distinct locations may not be rationally explained by comorbidity or disease progression in this case. Additionally, it is unclear whether the presentation could be ascribed to coincidence or the new *HTRA1* gene mutation. Based on the available studies, the imagine features vary among different families in CARASIL, and the underlying mechanisms are unclear. Besides disease progression, different mutation sites may also a cause of differential imaging findings. A case in point is the various degrees and locations of microbleeds and white matter hyperintensities found in patients with different gene mutations (2, 3, 15). In some families, microbleeds were absent, while most families showed mild microbleeds in cortico-subcortica regions and thalamus. Even more, a few families exhibited extend regions were involvement, such as midbrain and pons. On the other hand, anterior temporal lobe immunity in a few familial cases, whereas contrary findings were observed in some other studies. However, there is no study on the relationship between gene loci and imaging features at present. Under these circumstances, accurate diagnosis may be difficult to establish based on imaging manifestations alone; therefore, non-neurological signs and proper molecular testing should be considered for an accurate diagnosis.

## Conclusion

As a gene-associated orphan disease, the incidence of CARASIL in the general population is low, and there is limited knowledge about it. Our case describes a new genetic mutation that is not listed in the Exome Aggregation Consortium database. Our data further imply that different mutations could possibly lead to different imaging findings. The imaging features and potential mechanism of their versatility should be further investigated to gain deeper insights into this condition.

## Data Availability Statement

The original contributions presented in the study are included in the article/Supplementary Material, further inquiries can be directed to the corresponding author/s.

## Ethics Statement

Written informed consent was obtained from the individual(s) for the publication of any potentially identifiable images or data included in this article.

## Author Contributions

GL and CW conceptualized the case report idea. LW and JY analyzed the case and drafted the manuscript for intellectual content. JZ, SL, JL, YH, and CL prepared the MRI scans and figure. All authors reviewed the manuscript and were involved inpatients' healthcare. All authors contributed to the article and approved it for publication.

## Conflict of Interest

The authors declare that the research was conducted in the absence of any commercial or financial relationships that could be construed as a potential conflict of interest.

## Publisher's Note

All claims expressed in this article are solely those of the authors and do not necessarily represent those of their affiliated organizations, or those of the publisher, the editors and the reviewers. Any product that may be evaluated in this article, or claim that may be made by its manufacturer, is not guaranteed or endorsed by the publisher.

## References

[1] Hara K Shiga A Fukutake T Nozaki H Miyashita A Yokoseki A. Association of HTRA1 mutations and familial ischemic cerebral small-vessel disease. N Engl J Med. (2009) 360:1729–39. 10.1056/NEJMoa0801560 19387015

[2] Nozaki H Sekine Y Fukutake T Nishimoto Y Shimoe Y Shirata A. Characteristic features and progression of abnormalities on MRI for CARASIL. Neurology. (2015) 85:459–63. 10.1212/WNL.0000000000001803 26138950

[3] Nozaki H Nishizawa M Onodera O. Features of cerebral autosomal recessive arteriopathy with subcortical infarcts and leukoencephalopathy. Stroke. (2014) 45:3447–53. 10.1161/STROKEAHA.114.004236 25116877

[4] Uemura M Nozaki H Kato T Koyama A Sakai N Ando S. HTRA1-related cerebral small vessel disease: a review of the literature. Front Neurol. (2020) 11:545. 10.3389/fneur.2020.00545 32719647 PMC7351529

[5] Nozaki H Kato T Nihonmatsu M Saito Y Mizuta I Noda T. Distinct molecular mechanisms of HTRA1 mutants in manifesting heterozygotes with CARASIL. Neurology. (2016) 86:1964–74. 10.1212/WNL.0000000000002694 27164673

[6] Bianchi S Di Palma C Gallus GN Taglia I Poggiani A Rosini F. Two novel HTRA1 mutations in a European CARASIL patient. Neurology. (2014) 82:898–900. 10.1212/WNL.0000000000000202 24500651

[7] Preethish-Kumar V Nozaki H Tiwari S Vengalil S Bhat M Prasad C. CARASIL families from India with 3 novel null mutations in the HTRA1 gene. Neurology. (2017) 89:2392–4. 10.1212/WNL.0000000000004710 29101275

[8] Lee YC Chung CP Chao NC Fuh JL Chang FC Soong BW. Characterization of heterozygous HTRA1 mutations in Taiwanese patients with cerebral small vessel disease. Stroke. (2018) 49:1593–601. 10.1161/STROKEAHA.118.021283 29895533

[9] Menezes Cordeiro I Nzwalo H Sá F Ferreira RB Alonso I Afonso L. Shifting the CARASIL paradigm: report of a non-Asian family and literature review. Stroke. (2015) 46:1110–2. 10.1161/STROKEAHA.114.006735 25712943

[10] Ibrahimi M Nozaki H Lee A Onodera O Reichwein R Wicklund M. A CARASIL patient from Americas with novel mutation and atypical features: case presentation and literature review. Cerebrovasc Dis. (2017) 44:135–40. 10.1159/000477358 28628911

[11] Mendioroz M Fernández-Cadenas I Del Río-Espinola A Rovira A Solé E Fernández-Figueras MT. A missense HTRA1 mutation expands CARASIL syndrome to the Caucasian population. Neurology. (2010) 75:2033–5. 10.1212/WNL.0b013e3181ff96ac 21115960

[12] Liu J Dong F Hoh J. Loss of HtrA1-induced attenuation of TGF-beta signaling in fibroblasts might not be the main mechanism of CARASIL pathogenesis. Proc Natl Acad Sci USA. (2015) 112:E1693. 10.1073/pnas.1500911112 25770224 PMC4394265

[13] Fasano A Formichi P Taglia I Bianchi S Di Donato I Battisti C. HTRA1 expression profile and activity on TGF-beta signaling in HTRA1 mutation carriers. J Cell Physiol. (2020) 235:7120–7. 10.1002/jcp.29609 32017060

[14] Pantoni L. Cerebral small vessel disease: from pathogenesis and clinical characteristics to therapeutic challenges. Lancet Neurol. (2010) 9:689–701. 10.1016/S1474-4422(10)70104-6 20610345

[15] Verdura E Hervé D Scharrer E Amador Mdel M Guyant-Maréchal L Philippi A. Heterozygous HTRA1 mutations are associated with autosomal dominant cerebral small vessel disease. Brain. (2015) 138:2347–58. 10.1093/brain/awv155 26063658

