# Supplementary material for: Case Report: Diffuse Cerebral Microbleeds in Cerebral Autosomal Recessive Arteriopathy With Subcortical Infarcts and Leukoencephalopathy
Source: Front Neurol. 2022 Feb 9;13:818332. doi: 10.3389/fneur.2022.818332 (PMC8869253; doi:10.3389/fneur.2022.818332)

Case Report: Massive Cerebral Microbleeds in Cerebral Autosomal Recessive Arteriopathy With Subcortical Infarcts and Leukoencephalopathy

Lan Wen[1,2†], Jichao Yuan[2†], Shuang Li[3], Jieyi Zhao[1], Congjun Li[1], Jiafei Li[1], Yuanyuan Han[1], Chaohua Wang[1*], and Guangjian Li[2*]

[1]Department of Neurosurgery, West China Hospital, Sichuan University, Chengdu, China.

[2]Department of Neurology, Southwest Hospital, Third Military Medical University (Army Medical University), Chongqing, China.

[3]Department of Neurology, The Affiliated Hospital of Southwest Medical University, Luzhou, China.

† These authors have contributed equally to this work and share first authorship

*** Correspondence:**
Guangjian Li
lgjian991139@163.com

Chaohua Wang
neuro_chaohua_wang@163.com

Figure 1S: Gene sequencing. Targeted panel-sequencing revealed a novel homozygous missense mutation (c.508A > C, p.N170H) in HTRA1 of the patient, and her father had a heterozygous mutation at this site.

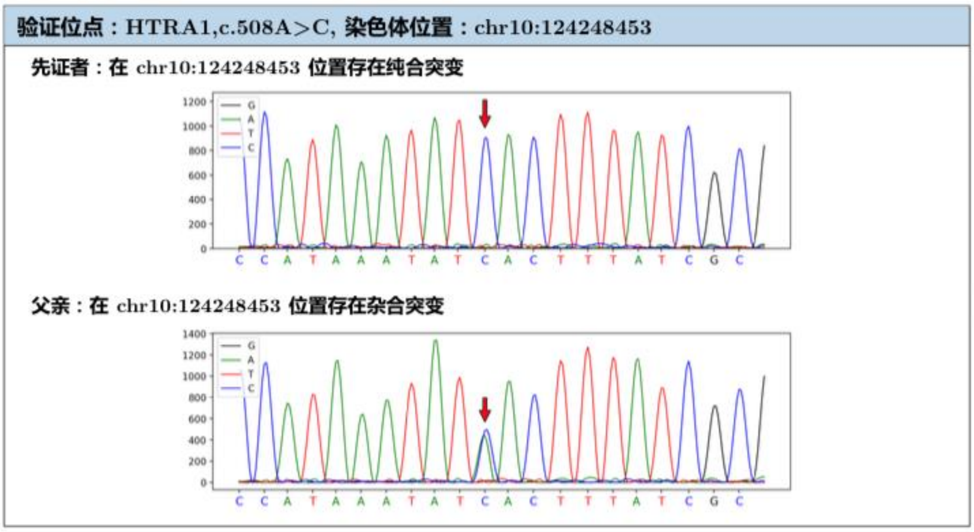

Figure 2S: This figure showed female pattern hair loss of the patient.

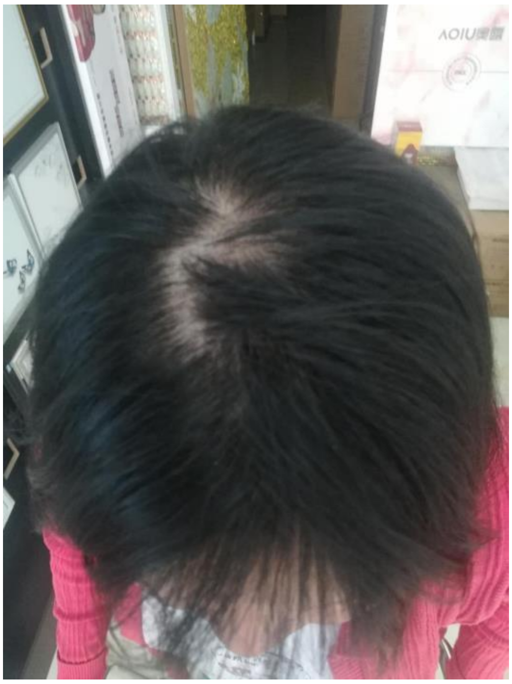

Supplement: Supplementary file 1 [file Data_Sheet_1.pdf]
